# A mutation in the low voltage-gated calcium channel *CACNA1G* alters the physiological properties of the channel, causing spinocerebellar ataxia

**DOI:** 10.1186/s13041-015-0180-4

**Published:** 2015-12-29

**Authors:** Hiroyuki Morino, Yukiko Matsuda, Keiko Muguruma, Ryosuke Miyamoto, Ryosuke Ohsawa, Toshiyuki Ohtake, Reiko Otobe, Masahiko Watanabe, Hirofumi Maruyama, Kouichi Hashimoto, Hideshi Kawakami

**Affiliations:** Department of Epidemiology, Research Institute for Radiation Biology and Medicine, Hiroshima University, Hiroshima, 1-2-3, Kasumi, Minami-ku, Hiroshima 734-8553 Japan; Laboratory for Cell Asymmetry, RIKEN Center for Developmental Biology, Kobe, Japan; Department of Neurology, Tokyo Metropolitan Health and Medical Treatment Corporation Ebara Hospital, Tokyo, Japan; Clinical and Molecular Genetics, Hiroshima University Hospital, Hiroshima, Japan; Department of Anatomy, Hokkaido University Graduate School of Medicine, Sapporo, Japan; Department of Clinical Neuroscience & Therapeutics, Graduate School of Biomedical and Health Sciences, Hiroshima University, Hiroshima, Japan; Department of Neurophysiology, Graduate School of Biomedical and Health Sciences, Hiroshima University, Hiroshima, Japan

**Keywords:** Spinocerebellar ataxia, *CACNA1G*, T-type calcium channel, Ca_V_3.1, Induced pluripotent stem cell

## Abstract

**Background:**

Spinocerebellar ataxia (SCA) is a genetically heterogeneous disease. To date, 36 dominantly inherited loci have been reported, and 31 causative genes have been identified.

**Results:**

In this study, we analyzed a Japanese family with autosomal dominant SCA using linkage analysis and exome sequencing, and identified *CACNA1G*, which encodes the calcium channel Ca_V_3.1, as a new causative gene. The same mutation was also found in another family with SCA. Although most patients exhibited the pure form of cerebellar ataxia, two patients showed prominent resting tremor in addition to ataxia. Ca_V_3.1 is classified as a low-threshold voltage-dependent calcium channel (T-type) and is expressed abundantly in the central nervous system, including the cerebellum. The mutation p.Arg1715His, identified in this study, was found to be located at S4 of repeat IV, the voltage sensor of the Ca_V_3.1. Electrophysiological analyses revealed that the membrane potential dependency of the mutant Ca_V_3.1 transfected into HEK293T cells shifted toward a positive potential. We established induced pluripotent stem cells (iPSCs) from fibroblasts of the patient, and to our knowledge, this is the first report of successful differentiation from the patient-derived iPSCs into Purkinje cells. There was no significant difference in the differentiation status between control- and patient-derived iPSCs.

**Conclusions:**

To date, several channel genes have been reported as causative genes for SCA. Our findings provide important insights into the pathogenesis of SCA as a channelopathy.

**Electronic supplementary material:**

The online version of this article (doi:10.1186/s13041-015-0180-4) contains supplementary material, which is available to authorized users.

## Background

Spinocerebellar ataxia (SCA) is characterized by cerebellar ataxia, dysarthria, oculomotor disorder, extrapyramidal sign, motor neuron sign, epilepsy, cognitive impairment, and many other symptoms [1]. To date, 36 loci have been shown to be involved in autosomal dominant SCA, and the causative genes and mutations have been identified for 31 types of SCAs [2]. The majority of SCAs account for repeat expansion, including a coding CAG repeat (SCA1–3, 6, 7, 17, and dentatorubral-pallidoluysian atrophy) causing misfolding of the encoded protein due to the expanded polyglutamine tracts; groups of noncoding CAG, CTG, ATTCT, and GGCCTG repeats (SCA8, 10, 12, and 36); and large TGGAA repeat insertions (SCA31) that induce RNA-mediated gain-of-function mechanisms. The remaining known SCA types are caused by single nucleotide variants or indels (SCA5, 11, 13–15, 18–21, 23, 26–28, 34, 35, 38, 40, and 41) in genes that encode various functional proteins [2].

*CACNA1A* (MIM 601011) was first reported as a causative gene of SCA6 (MIM 183086) among the genes encoding channels [3, 4]. In recent years, potassium channel mutations have been described in SCA13 (MIM 605259) and SCA19/22 (MIM 607346) [5–7]. Episodic ataxia (EA) is one of the diseases associated with SCA, and some types of EA are caused by mutations in calcium channel. *CACNA1A* and *CACNB4* mutations lead to EA2 (MIM 108500) and EA5 (MIM 613855), respectively [8, 9]. Thus the ion channel dysfunction plays a key role in the pathogenesis of ataxia and related diseases.

We have been engaged in clinicogenetic research using the samples from more than 2,000 patients with SCA. From a thorough examination of the disease types and the geographical distributions of the 2,121 patients, causative genes for 26.6 % of the dominant-inherited cases (205 out of 721) are still unknown [10]. Therefore, in order to identify novel causative genes, we applied exome sequencing to the families with dominantly inherited SCA.

## Methods

### Patients

**Fig. 1:**
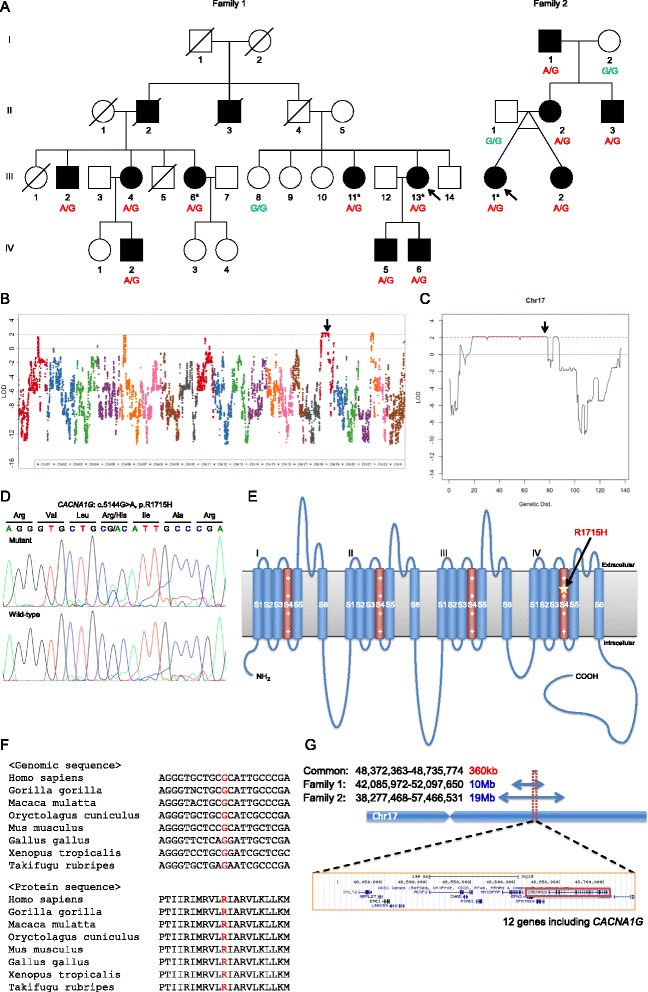
Identification of a mutation in *CACNA1G* causing SCA. **a** Pedigree charts of families 1 and 2. Arrows indicate the probands. Filled and open symbols represent affected and unaffected individuals, respectively. Genotypes of the variant c.5144G > A are shown under the number of samples. Asterisks indicate the patients used for exome sequencing. **b**, **c** Results of linkage analysis. Arrows indicate the positions of *CACNA1G*. **d** Sanger sequencing to confirm the *CACNA1G* variant. The reference nucleotide G is overlapped with variant nucleotide A in the mutant sample. **e** Structure of Ca_V_3.1 encoded by *CACNA1G*. The star indicates the position of the identified mutation. The mutation was located in the segment 4 (S4) of the fourth repeat. **f** Conservation at the location of the mutation. The nucleotide and amino acid sequences are completely conserved among vertebrates. **g** Haplotype analysis. From the result of SNP genotyping, the haplotypes of both families around the *CACNA1G* gene coincided for 360 kb

We enrolled two Japanese families with segregating dominant traits for cerebellar ataxia. There are 10 affected individuals in the family 1 and five in the family 2. Blood samples were obtained from eight affected individuals and three unaffected individuals in family 1, and five affected individuals and two unaffected individuals in family 2 (Fig. 1a). All patients were diagnosed with SCA by neurologists. Prior to this study, we confirmed that all affected individuals had no pathogenic mutations causing SCA1–3, 6, 8, and dentatorubral-pallidoluysian atrophy. The study was approved by the Human Subjects Committees of Hiroshima University; all subjects provided written informed consent.

### Linkage analysis

The samples used for linkage analysis were 1-III-2, 1-III-4, 1-III-6, 1-III-8, 1-III-11, 1-III-13, 1-IV-2, 1-IV-3, and 1-IV-4. Because it was possible that 1-IV-3 and 1-IV-4 did not reach the appropriate age at onset, the two samples were treated as unknown in the pedigree file of linkage analysis. Genomic DNA (gDNA) was extracted from the peripheral lymphocytes of the participants according to standard protocols. We used a Genome-Wide Human SNP Array 6.0 (Affymetrix, Santa Clara, CA, USA) for genotyping of single nucleotide polymorphisms (SNPs), and linkage analysis was performed by Allegro software, estimating the dominant inheritance [11].

### Exome Sequencing

Exome sequencing was carried out using three samples from 1-III-6, 1-III-11, and 1-III-13, as previously described [12]. For family 2, exome sequencing was also performed with the sample from 2-III-1. gDNA libraries were prepared using a SeqCap EZ Human Exome Library v2.0 (Roche, Basel, Switzerland). Sequencing was performed with 100-bp paired-end reads on a HiSeq2000 sequencer (Illumina, San Diego, CA, USA). We used BWA (http://bio-bwa.sourceforge.net/) [13] for alignment and mapping, Samtools (http://samtools.sourceforge.net/) [14] and Picard (http://broadinstitute.github.io/picard/) for SAM/BAM handling, GATK (http://www.broadinstitute.org/gatk/) [15] and Samtools for variant calls, and Annovar (http://annovar.openbioinformatics.org/) [16] for annotation. Functional predictions due to amino acid changes were estimated using PolyPhen-2 (http://genetics.bwh.harvard.edu/pph2/) [17], SIFT (http://sift.bii.a-star.edu.sg/) [18], and Mutation Taster (http://www.mutationtaster.org/index.html) [19]. Control exome sequences were obtained from Japanese patients undergoing exome analysis for diseases other than SCA. All reported genomic coordinates were in GRCh37/hg19. The identified mutations were validated with a standard polymerase chain reaction (PCR)-based amplification followed by sequence analysis with an Applied Biosystems 3130 DNA sequencer (Thermo Fisher Scientific, Waltham, MA, USA).

### Expression vector

Wild-type *CACNA1G* (short isoform; BC110995.1, NM_198382.2) in the pCMV-SPORT6 plasmid (pCMV-SPORT6-CACNA1G) was purchased from Dharmacon (Lafayette, CO, USA). The mutation c.5075G > A corresponding to c.5144G > A in the longest isoform (NM_018896.4) was introduced by site-directed mutagenesis using QuikChange Lightning (Agilent Technologies, Santa Clara, CA, USA) and verified by bidirectional sequencing. The IRES-EGFP sequence was amplified by PCR from the pIRES-EGFP plasmid and inserted at the termination codon of the cDNA sequence in pCMV-SPORT6-CACNA1G (pCMV-SPORT6-CACNA1G-IG) using an In-Fusion HD Cloning Kit (TaKaRa Bio, Shiga, Japan).

### Cell culture, transfection, and immunofluorescence

The primary antibodies used in this study were anti-CACNA1G [20], and anti-alpha 1 sodium potassium ATPase (Abcam, Cambridge, UK).

HeLa and HEK293T cells were maintained in Dulbecco’s modified Eagle’s medium (DMEM; Nakarai Tesque, Kyoto, Japan) supplemented with 10 % fetal bovine serum and penicillin/streptomycin (PS) in a 37 °C incubator with 5 % CO_2_. For immunofluorescence analysis, cells were grown on chamber slides (SCS-008; Matsunami, Osaka, Japan) coated with poly-l-lysine (Sigma-Aldrich, St. Louis, MO, USA), and were transiently transfected with pCMV-SPORT6-CACNA1G using Lipofectamine LTX (Thermo Fisher Scientific, Waltham, MA, USA), according to the manufacturer’s instructions. After 48–72 h, the cells were fixed in 4 % paraformaldehyde, washed with phosphate-buffered saline (PBS), blocked, and permeabilized with 0.2 % Tween20. Cells were incubated overnight at 4 °C with anti-CACNA1G and anti-alpha 1 sodium potassium ATPase antibodies and then treated with secondary antibodies. Images were obtained using confocal microscopy (LSM510; Carl Zeiss, Jena, Germany). The nuclei were visualized using DAPI.

Cells for whole-cell patch clamping were grown in glass-bottom plates (μ-Dish 35 mm low; ibidi, Martinsried, Germany) for 24 h following transfection with SPORT6-CACNA1G-IG using Lipofectamine LTX.

### Electrophysiology

Whole-cell recordings were made from GFP-expressing HEK293T cells using an upright microscope (BX51WI; Olympus, Tokyo, Japan) equipped with an IR-CCD camera system (IR-1000; DAGE-MTI, Michigan, IN, USA) at room temperature. The intracellular solution was composed of 110 mM CsCl, 20 mM TEA-Cl, 10 mM NaCl, 5 mM EGTA, 10 mM HEPES, 4 mM Mg-ATP, and 0.4 mM 2Na-GTP (pH 7.3, adjusted with CsOH). The pipette access resistance was about 2–3 MΩ. The composition of the extracellular solution was 10 mM NaCl, 105 mM TEA-Cl, 10 mM 4-AP, 2.5 mM KCl, 2 mM CaCl_2_, 1 mM MgSO_4_, 1.25 mM NaH_2_PO_4_, 26 mM NaHCO_3_, and 20 mM glucose, bubbled with 95 % O_2_ and 5 % CO_2_. Ionic currents were recorded with an EPC-10 (HEKA Elektronik, Lambrecht, Germany). The signals were filtered at 3 kHz and digitized at 20 kHz. On-line data acquisition and off-line data analysis were performed using PATCHMASTER software (HEKA Elektronik, Lambrecht, Germany). Relative conductance and steady-state inactivation plots were fitted by the following Boltzmann equations:

$$ \begin{array}{ll}\frac{G}{G_{max}}\hfill & =\frac{1}{1+ exp\left(\left({V}_{half}-{V}_m\right)/k\right)}\hfill \\ {}\frac{I}{I_{max}}\hfill & =\frac{1}{1+ exp\left(\left({V}_m-{V}_{half}\right)/k\right)}\hfill \end{array} $$

### Derivation of patient-specific fibroblasts and generation of induced pluripotent stem cells (iPSCs)

Patient 2-III-1 harbored the *CACNA1G* mutation. Thus, a 3-mm punched skin biopsy was obtained from the upper arm. The skin sample was mechanically cut into small pieces and cultured in 100-mm cell culture dishes in DMEM containing 20 % fetal bovine serum. Outgrowth of dermal fibroblasts appeared after 7–14 days, and cultures were split at 1:3 after reaching 80 % confluence. Peripheral blood was obtained from a healthy donor after providing written informed consent in accordance with the institutional review board guidelines. Peripheral blood mononuclear cells (PBMCs) were purified by density gradient centrifugation with a BD Vacutainer CPT (BD Biosciences, Franklin Lakes, NJ, USA) according to the manufacturer’s instructions.

Human complementary DNAs for reprogramming factors were transduced into dermal fibroblasts and PBMCs with episomal vectors (pCXLE-hOCT3/4-shp53-F, pCXLE-hSK, and pCXLE-hUL; Addgene, Cambridge, MA, USA) [21, 22]. The generated iPSCs were maintained on a mitomycin C-inactivated SNL feeder cell layer in DMEM/F12 containing GlutaMaxI (Thermo Fisher Scientific, Waltham, MA, USA) supplemented with 20 % KnockOut Serum Replacement (KSR; Thermo Fisher Scientific, Waltham, MA, USA), 5 ng/ml recombinant human FGF basic (Wako, Osaka, Japan), 0.1 mM β-mercaptoethanol (Sigma-Aldrich, St. Louis, MO, USA) and PS under 5 % CO_2_.

### Purkinje cell differentiation from iPSCs

The differentiation of Purkinje cells from iPSCs was performed as described previously [23, 24].

## Results

### Clinical manifestations

The proband 1-III-13 has exhibited the pure form of cerebellar ataxia, and her age of at disease onset was 45 years. The main symptoms were ataxic gait, dysarthria, and gaze-evoked horizontal nystagmus. Cognitive impairment, epilepsy, muscle atrophy, involuntary movement, and parkinsonism were not observed. The parents did not exhibit the same symptoms; however, symptoms were observed in her uncles. Her sister (1-III-11) also suffered from the same symptoms, with slightly increased severity. Other affected members in the family 1 showed similar clinical features, and the age at onset varied from 20 to 70 years. In all patients, progression of the disease was relatively slow.

For family 2, the age at onset ranged from 18 to 57 years. The main symptoms and clinical course were similar to those in family 1, except that two patients 2-II-2 and 2-III-2 showed prominent resting tremors.

In neuroimaging, brain magnetic resonance imaging (MRI) results of 2-III-1 demonstrated cerebellar atrophy, typical of SCA. However, the cerebrum and brainstem showed no abnormalities (Additional file 1: Figure S1).

### Candidate region from linkage analysis

From the results of linkage analysis, two regions where logarithm of odds (LOD) score was 2 or more were obtained in chromosomes 17 and 21 (Fig. 1b). The maximum LOD scores for chromosomes 17 and 21 were 2.1067 and 2.1066, respectively. The candidate region on chromosome 17 was relatively wide, approximately 60 cM (Fig. 1c). The region did not correspond with any genetic loci of known SCAs.

### Variant filtration

The results of exome sequencing are shown in Additional file 2: Table S1. More than 90,000 variants in each sample were called. We narrowed down the candidate variants based on filtering criteria consisting of open databases (dbSNP, 1000 Genomes, and EPS 6400), genomic position, function, and zygosity. A total of 386 variants were common to 1-III-6, 1-III-11, and 1-III-13. Among them, we excluded variants that were observed in other diseases from our in-house variant database, and 11 heterozygous variants remained (Additional file 3: Table S2).

Variants that were estimated to be pathogenic by two or more of the three prediction algorithms were as follows: *SLC15A1*, *ALOX15B*, *CACNA1G*, and *RNF213*. We performed segregation of the variants with the disease in the 11 available subjects from family 1. The variants in *ALOX15B* and *CACNA1G* were observed only in affected subjects, but not in unaffected subjects. Based on the linkage analysis, the variant of *CACNA1G* was present in the high LOD region; therefore, we concluded that *CACNA1G* was the most likely candidate. This variant was confirmed by Sanger sequencing (Fig. 1d).

### Heterozygous mutation in *CACNA1G*

Chr17:48694921G > A corresponded to *CACNA1G*:c.5144G > A, p.Arg1715His (NM_018896.4, MIM 604065) (Fig. 1d). The *CACNA1G* gene encodes the Ca_V_3.1 T-type voltage-dependent calcium channel (VDCC). The nucleotide and amino acid sequences of the mutated region were found to be completely conserved among vertebrates (Fig. 1f). The structure of the calcium channel α subunit, including Ca_V_3.1, consisted of four repeating domains (repeats I–IV) comprised of five hydrophobic segments (S1–3, S5, and S6) and one positively charged segment (S4) each [25]. The identified mutation was located in S4 of repeat IV (Fig. 1e).

Identical heterozygous mutation was found in the variants obtained by exome sequencing for the sample from 2-III-1 of family 2. No other variants that caused SCA were found in this patient. Sequencing of *CACNA1G* throughout family 2 revealed that affected members harbored the mutation and unaffected members did not. From the results of SNP genotyping performed for linkage analysis, we compared the haplotypes around the *CACNA1G* gene in the two families. Families 1 and 2 shared the haplotypes in the range of 10 and 19 Mb, respectively. Both families shared the haplotypes for a length of 360 kb (Fig. 1g).

### Localization of mutant Ca_V_3.1

Immunocytochemical studies were performed in order to examine the possible changes in the subcellular localization caused by the mutation. We used the NaK-ATPase as a plasma membrane marker. Both wild-type and mutant Ca_V_3.1 were distributed diffusely in the cytoplasm and the plasma membrane (Additional file 4: Figure S2). No significant differences were observed in the localizations of wild-type and mutant Ca_V_3.1.

### Electrophysiological properties

We examined changes in the electrophysiological properties of mutant Ca_V_3.1. Whole-cell recording was obtained from GFP-positive HEK293T cells expressing wild-type or mutant Ca_V_3.1. To isolate Ca^2+^ currents, HEK293T cells were bathed with the extracellular solution containing potassium channel blockers.

**Fig. 2:**
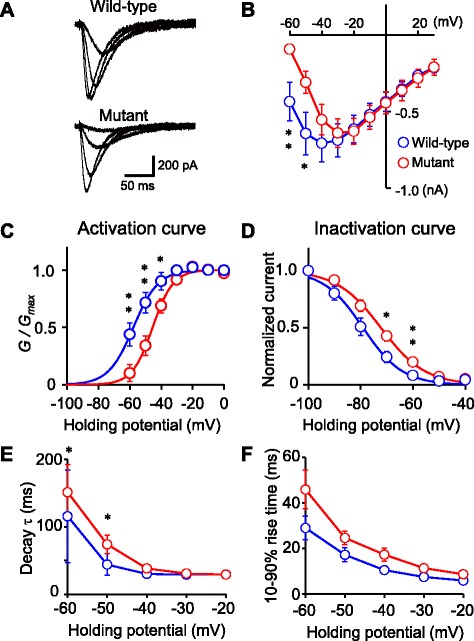
Changes in electrophysiological properties. **a** Representative traces of T-type VDCC currents recorded from HEK293T cells expressing wild-type (upper) or mutant (lower) Ca_V_3.1. Holding potential was -60 mV. Voltage steps were applied after a hyperpolarizing prepulse to -100 mV (duration = 500 ms). **b** Peak current-voltage plots of VDCC currents of wild-type (blue, *n* = 9) and mutant (red, *n* = 12) Ca_V_3.1. **c** Relative conductance-voltage plots. Each data point was calculated from data in B. The black line is a fit using the Boltzmann equation (see Methods). **d** Steady state inactivation-voltage plots. The voltage step to -30 mV was preceded by incremental hyperpolarizing pulses (duration = 300 ms). Data were obtained from the same cells shown in B and C. The black line is a fit using the Boltzmann equation (see Methods). **e** The decay time constants of inactivation of the Ca^2+^ current plotted against voltage steps. **f** The 10–90 % rise times of Ca^2+^ currents plotted against voltage steps. Data are presented as the mean ± SEM. Statistical significance was assessed by Mann-Whitney *U*-test. **p < 0.01, *p < 0.05

T-type VDCC currents were elicited by depolarizing voltage steps after a hyperpolarizing prepulse to -100 mV (300 ms) in HEK293T cells carrying wild-type and mutant constructs (Fig. 2a). The peak current amplitudes are plotted against step voltages in Fig. 2b. In HEK293T cells expressing the mutant construct, amplitudes of T-type VDCC currents were significantly smaller at step voltages of -60 and -70 mV, but not at higher membrane potential depolarizations (Fig. 2a). This result suggested that the maximum conductance was normal, but that activation of mutant Ca_V_3.1 was shifted to depolarized membrane potentials. To further examine this phenomenon, the, estimated relative conductances were calculated from the peak current-voltage relationships. The reversal potentials used for correction were estimated by line fits of peak amplitudes from -10 to +30 mV. For each cell, the relative conductance was plotted against depolarizing voltage pulses and fitted by a Boltzmann equation (Fig. 2c). The averaged half-conductance potential of -57.5 ± 9.1 mV in wild-type (n = 9, mean ± SD) was more hyperpolarized than that of -44.7 ± 6.0 mV in mutant (n = 12; *p* = 0.004, Mann-Whitney *U*-test), suggesting that activation of mutant Ca_V_3.1 was significantly shifted toward more positive membrane potentials. In contrast, the slope factor was not significantly different (wild-type, 6.4 ± 2.2 mV; mutant, 6.2 ± 2.9 mV; *p* = 0.303), suggesting parallel shift of membrane potential dependent curve to positive potentials. Steady-state inactivation was calculated by fitting the relative amplitudes of the currents as a function of the hyperpolarizing prepulse with the Boltzmann equation (Fig. 2d). Similar to activation, the half-steady-state inactivation potentials were significantly shifted to more positive membrane potentials (wild-type, -79.1 ± 6.6 mV; mutant, -72.5 ± 5.5 mV; *p* < 0.025), but the slope factor was not significantly different (wild-type, 6.3 ± 1.4 mV; mutant, 7.7 ± 1.5 mV; *p* = 0.07). As a consequence, the window current shifted toward more positive membrane potentials. The kinetics of the T-type VDCC current were also affected. The inactivation decay time constant of the T-type VDCC currents was significantly slower at small voltage steps in HEK293T cells expressing the mutant construct (Fig. 2e). While it was not statistically significant, a similar trend was also observed for the 10–90 % rise time (Fig. 2f). These lines of evidence suggested that the mutation affected the voltage dependency of activation and inactivation of the Ca_V_3.1 T-type VDCCs.

### Purkinje cells derived from iPSCs

**Fig. 3:**
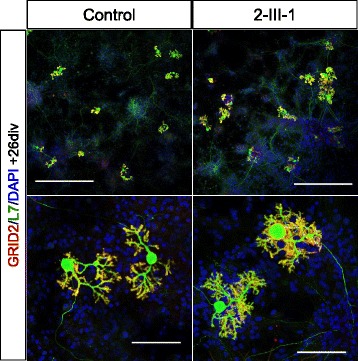
iPSC-derived Purkinje cells. Immunostaining of iPSC-derived Purkinje cells on co-culture days 26. GRID2 was expressed in the L7^+^ dendritic spines. The scale bars represent 500 μm (upper panels) and 100 μm (lower panels)

We previously reported that the cerebellar neuroepithelium self-organizes in 3D human embryonic stem cell culture. The self-organized cerebellar neuroepithelium differentiates into mature Purkinje cells [24]. To examine the ability of the generated iPSCs to differentiate into Purkinje cells, we applied the 3D system to iPSC culture. The differentiated iPSC-derived cells expressed L7. The L7^+^ cells developed elaborate dendritic branches and spines that were positive for Purkinje cell-specific glutamate receptor GRID2 (Fig. 3, total culture days 51–58). From the result of morphological evaluation of differentiated Purkinje cells, dendritic field areas were 14,367 ± 1,026 μm^2^ in control (n = 6, mean ± SD) and 12,287 ± 954.8 μm^2^ in patient-derived iPSC (n = 6, mean ± SD; Additional file 5: Figure S3A), and diameters of cell soma were 29.8 ± 0.55 μm in control (n = 7, mean ± SD) and 30.09 ± 2.22 μm in patient-derived (n = 7, mean ± SD; Additional file 5: Figure S3B). There were no obvious differences in the differentiation potentials between control- and patient-derived neurons immunocytochemically and morphologically.

## Discussion

Our study revealed that a mutation in *CACNA1G* encoding the T-type calcium channel Ca_V_3.1 causes SCA. This finding is consistent with the fact that several mutations in channel-encoding genes have been reported to cause SCA. We identified the mutation from a large Japanese family with autosomal dominant SCA using exome sequencing and linkage analysis. Moreover, the same mutation was found in another Japanese family with SCA.

The mutation was located in the S4 segment, which is assumed as a part of the voltage sensor. In particular, the substituted arginine is one of the positively charged residues found every three amino acids; these residues play a central role in voltage sensing. Since simultaneous activation of all four voltage sensors is required to open the calcium channel, the arginine residue mutated in the affected individuals is electrophysiologically important [26]. Our electrophysiological analysis supported this concept. While the subcellular distribution and membrane translocation of the protein were normal, membrane potential dependency of activation and inactivation curves of Ca^2+^ currents were positively shifted in HEK293T cells expressing mutant Ca_V_3.1. Very recently, identical mutation in *CACNA1G* was reported in French families, which also demonstrated positive shifts in the window current of the T-type channel [27]. Alterations of T-type Ca^2+^ currents were consistent with our results. The mild physiological changes could explain the clinical features of SCA, including the age at onset and slow progression of the disease.

Ca_V_3.1 is expressed in the cerebellum, inferior olive nucleus, and the thalamus [28]. Physiologically, the channel is associated with high-frequency burst firing of more than 10 Hz and oscillation [29, 30] and regulates the extension of the axons and dendrites in the developmental stage [31]. In animal models, Ca_V_3.1 has been implicated in epilepsy. Ca_V_3.1 knockout mice are unable to elicit thalamocortical low-threshold calcium potentials (LTCPs) and burst firing in response to hyperpolarization and are resistant to drug-induced absence seizures [32]. Conversely, overexpression of Ca_V_3.1 in transgenic mice induces thalamocortical spike-wave oscillations characteristic of pure absence epilepsy [33]. LTCPs of the thalamus are also important for stabilizing NREM sleep [34]. In another study using Ca_V_3.1 knockout mice, Ca_V_3.1 expressed in the inferior olive was shown to be associated with tremor [30].

The clinical picture of our patients was similar to that of SCA6, caused by mutation in another type of calcium channel, *CACNA1A*. Most patients exhibited the pure form of cerebellar ataxia. Notably, two patients had resting tremors. Ca_V_3.1 is expressed at high levels in the cerebellum and inferior olive nucleus. Because T-type VDCCs have important roles in the rhythmogenesis of inferior olivery neurons [35], the symptom may also be the consequence of the Ca_V_3.1 mutation. Pyramidal tract signs, which are characteristic of the French families, were not prominent in our cases [27]. The fact that the same mutation in the *CACNA1G* gene was independently identified in the different ethnics, Japanese and French further supported the concept of *CACNA1G* as the causative gene for autosomal dominant SCA. Further studies are needed to clarify the clinical characteristics of the *CACNA1G*-dependent SCA in more detail.

In order to examine the role of *CACNA1G* in neurogenesis, iPSCs derived from the patient were differentiated into Purkinje cells. There were no significant defects in the morphology of Purkinje cells or the expression of Purkinje cell markers, and therefore, the mutation likely did not affect the differentiation of the cells into Purkinje cells. In the future, we are planning on conducting a functional evaluation of induced Purkinje cells.

## Conclusions

In conclusion, we identified a mutation in *CACNA1G* encoding the T-type calcium channel from two families with dominantly inherited SCA. The mutation is located in the crucial residue of the S4 segment, which functions as a voltage sensor. Our electrophysiological study corroborated these changes in channel properties, and thus, the mutation was likely to be the cause of SCA. We indicated the novel evidence that ion channel dysfunction leads to the pathogenesis of SCA.

## Additional files

Additional file 1: Figure S1. Brain MRI of the patient 2-III-1. The left panel shows the T1-weighted sagittal image, and the right panel shows the T1-weighted axial image. Marked cerebellar vermian atrophy was observed. (PPTX 1622 kb) Additional file 2: Table S1. Summary of exome sequencing. (XLSX 42 kb) Additional file 3: Table S2. Candidate variants. (XLSX 35 kb) Additional file 4: Figure S2. Confocal images of HeLa cells expressing wild-type or p.Arg1715His mutant Ca_V_3.1. HeLa cells were transiently transfected with wild-type or p.Arg1715His mutant human Ca_V_3.1 and immunostained with anti- Ca_V_3.1 antibodies (green) and anti-NaK-ATPase antibodies (red) as a membrane marker. Scale bar is 20 μm. (PPTX 286 kb) Additional file 5: Figure S3. Morphological measurement of differentiated Purkinje cells. Dendritic field area (A) and diameter of cell soma (B) were measured in differentiated Purkinje cells. Data are presented as the mean ± SD. Statistical significance was assessed by unpaired *t*-test. (PPTX 274 kb)

## Abbreviations

SCA: Spinocerebellar ataxia
EA: Episodic ataxia
gDNA: genomic DNA
SNP: Single nucleotide polymorphism
PCR: Polymerase chain reaction
iPSC: induced pluripotent stem cell
PBMC: Peripheral blood mononuclear cell
MRI: Magnetic resonance imaging
LOD: Logarithm of odds
VDCC: Voltage-dependent calcium channel
LTCP: Low-threshold calcium potential
NREM: Non-rapid eye movement
