# Supplementary material for: A mutation in the low voltage-gated calcium channel CACNA1G alters the physiological properties of the channel, causing spinocerebellar ataxia
Source: Mol Brain. 2015 Dec 29;8:89. doi: 10.1186/s13041-015-0180-4 (PMC4693440; doi:10.1186/s13041-015-0180-4)
Supplement: Additional file 1: Figure S1. — Brain MRI of the patient 2-III-1. The left panel shows the T1-weighted sagittal image, and the right panel shows the T1-weighted axial image. Marked cerebellar vermian atrophy was observed. (PPTX 1622 kb) [file 13041_2015_180_MOESM1_ESM.pptx]

## Slide 1
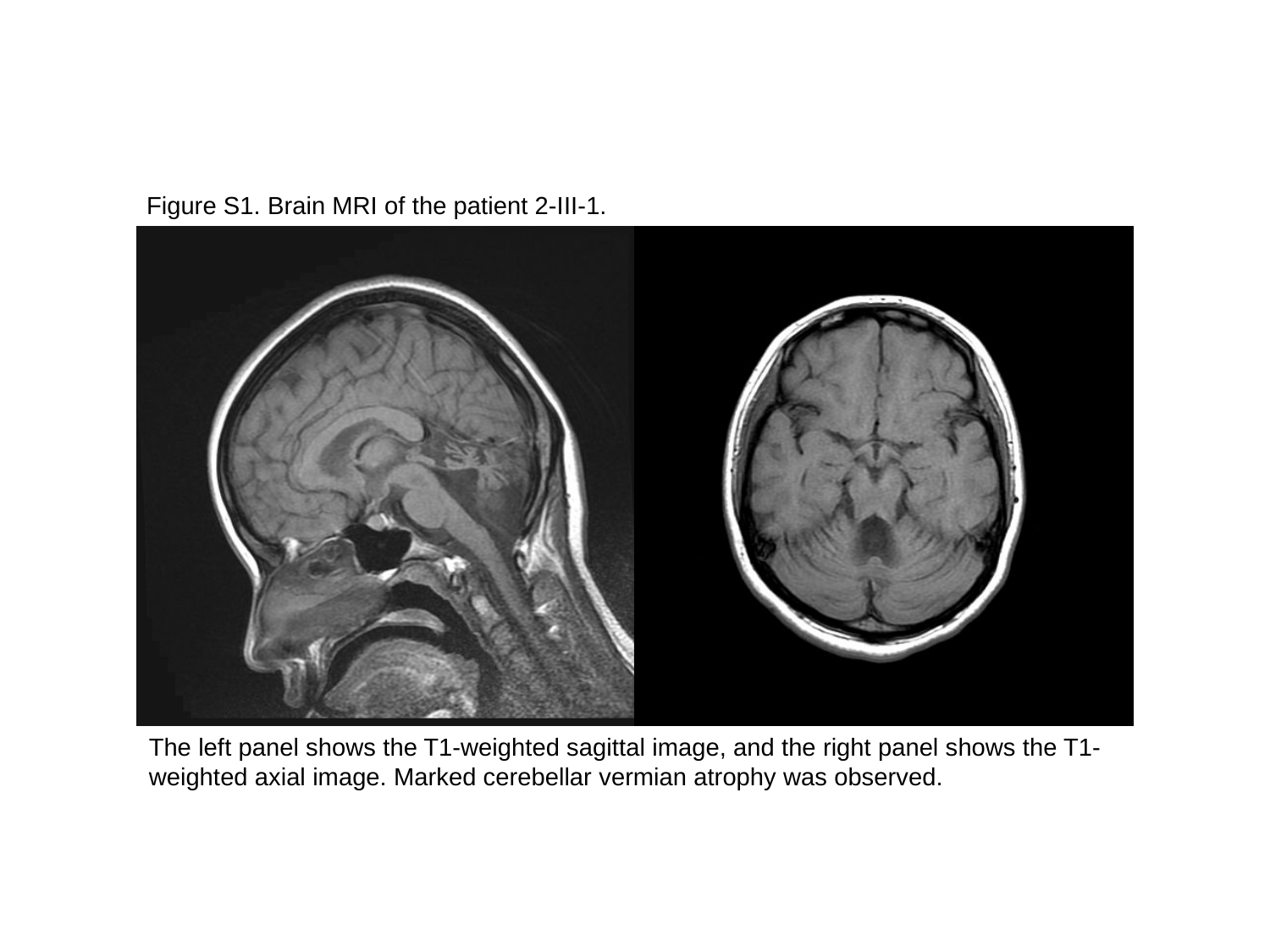

Figure S1. Brain MRI of the patient 2-III-1.
The left panel shows the T1-weighted sagittal image, and the right panel shows the T1-weighted axial image. Marked cerebellar vermian atrophy was observed.
