# Supplementary material for: A mutation in the low voltage-gated calcium channel CACNA1G alters the physiological properties of the channel, causing spinocerebellar ataxia
Source: Mol Brain. 2015 Dec 29;8:89. doi: 10.1186/s13041-015-0180-4 (PMC4693440; doi:10.1186/s13041-015-0180-4)
Supplement: Additional file 4: Figure S2. — Confocal images of HeLa cells expressing wild-type or p.Arg1715His mutant CaV3.1. HeLa cells were transiently transfected with wild-type or p.Arg1715His mutant human CaV3.1 and immunostained with anti- CaV3.1 antibodies (green) and anti-NaK-ATPase antibodies (red) as a membrane marker. Scale bar is 20 μm. (PPTX 286 kb) [file 13041_2015_180_MOESM4_ESM.pptx]

## Slide 1
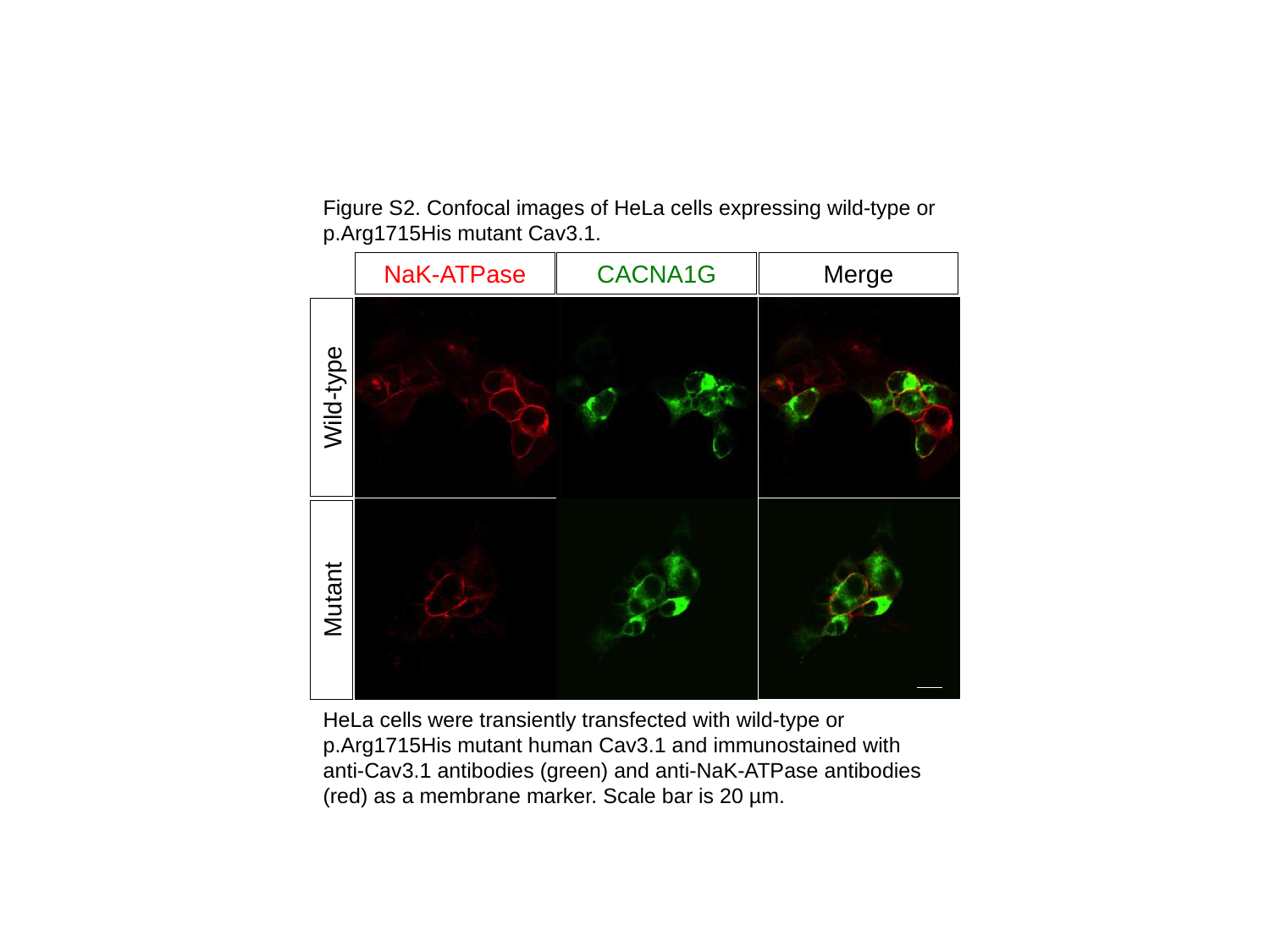

Figure S2. Confocal images of HeLa cells expressing wild-type or p.Arg1715His mutant Cav3.1.
NaK-ATPase
CACNA1G
Merge
Wild-type
Mutant
HeLa cells were transiently transfected with wild-type or p.Arg1715His mutant human Cav3.1 and immunostained with anti-Cav3.1 antibodies (green) and anti-NaK-ATPase antibodies (red) as a membrane marker. Scale bar is 20 µm.
