# Supplementary material for: A mutation in the low voltage-gated calcium channel CACNA1G alters the physiological properties of the channel, causing spinocerebellar ataxia
Source: Mol Brain. 2015 Dec 29;8:89. doi: 10.1186/s13041-015-0180-4 (PMC4693440; doi:10.1186/s13041-015-0180-4)
Supplement: Additional file 5: Figure S3. — Morphological measurement of differentiated Purkinje cells. Dendritic field area (A) and diameter of cell soma (B) were measured in differentiated Purkinje cells. Data are presented as the mean ± SD. Statistical significance was assessed by unpaired t-test. (PPTX 274 kb) [file 13041_2015_180_MOESM5_ESM.pptx]

## Slide 1
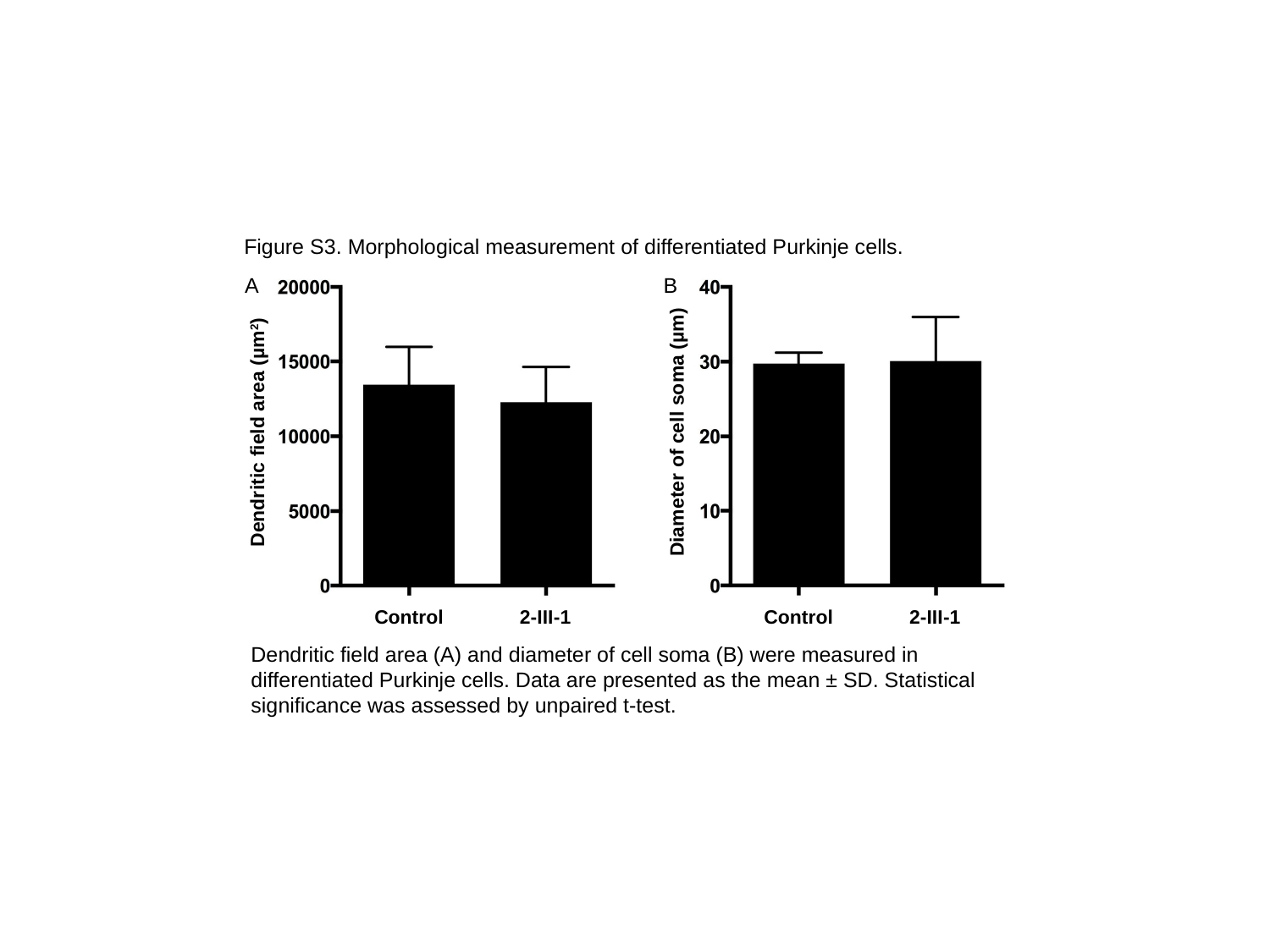

Figure S3. Morphological measurement of differentiated Purkinje cells.
A
Dendritic field area (µm2)
Control
2-III-1
B
Diameter of cell soma (µm)
Control
2-III-1
Dendritic field area (A) and diameter of cell soma (B) were measured in differentiated Purkinje cells. Data are presented as the mean ± SD. Statistical significance was assessed by unpaired t-test.
